# Defining MRI-based follow-up protocol for primary central nervous system lymphoma

**DOI:** 10.1007/s00277-024-06145-5

**Published:** 2024-12-19

**Authors:** Inka K. Puhakka, Kaisa L. Sunela, Aino L. Rönkä, Aino M. Rajamäki, Ulla-Mari Arkko, Tuula M. Klaavuniemi, Milla E.L Kuusisto, Pekka A. Jäkälä, Tuomas A. Selander, Hanne K. Kuitunen, Anne-Mari Kantanen, Outi M. Kuittinen

**Affiliations:** 1 Department of Neurology, Kuopio University Hospital, Puijonlaaksontie 2, 70210 Kuopio, Finland; 2 Department of Oncology, Tampere University Hospital, Tampere, Finland; 3 Department of Oncology, Kuopio University Hospital, Kuopio, Finland; 4 Department of Oncology, Hospital Nova of Central Finland, Jyväskylä, Finland; 5 Department of Oncology, North Karelia Central Hospital, Joensuu, Finland; 6 Department of Oncology, Mikkeli Central Hospital, Mikkeli, Finland; 7 Cancer Centre, Oulu University Hospital, Oulu, Finland; 8 Science Service Center, Kuopio University Hospital, Kuopio, Finland; 9 Oncology, Institute of Medical Medicine, Faculty of Health Medicine, University of Eastern Finland, Kuopio, Finland; 10 Neurology, Institute of Medical Medicine, Faculty of Health Medicine, University of Eastern Finland, Kuopio, Finland; 11 Biomedicine and Internal Medicine Research Unit, University of Oulu, Oulu, Finland; 12 Medical Reseach Unit, Oulu University Hospital, Oulu, Finland; 13 Department of Internal Medicine, Länsi-Pohja Central Hospital, Kemi, Finland; 14 Finnish Medicines Agency, Tampere, Finland

**Keywords:** PCNSL, Primary CNS lymphoma, Relapse, Follow-up, Imaging

## Abstract

**Background:**

The optimal follow-up protocol for primary central nervous system lymphoma (PCNSL) is unclear. This is the first study to evaluate the benefit of structured follow-up imaging of PCNSL with respect to the timing of relapse.

**Methods:**

A total of 198 PCNSL patients (57.1% males) diagnosed between 2003 and 2020 were included. The data were collected retrospectively from 8 hospitals in Finland. Relapse detection details included structured follow-up imaging (sMRI) studies, additional imaging (aMRI) studies based on patients’ new symptoms, and all outpatient and emergency visits. Overall survival (OS) with respect to the relapse detection method, sMRI versus aMRI, was also evaluated.

**Results:**

Relapse was diagnosed in 71 patients (35.9%), 66.1% of whom experienced relapse during the first 2 years after diagnosis. During the first year, 48.3% (14/29) of the relapses were detected via sMRI, and 51.7% (15/29) via aMRI. During the second year, the percentages were 33.3% and 66.7%, respectively. More than 5 years after the diagnosis, all the relapses were detected via aMRI. To observe one relapse during the first year, 9.4 sMRI studies were needed. Overall survival after relapse (OS2) was 4.0 months for the patients whose first relapse was detected via sMRI and 3.0 months for those whose first relapse was detected via aMRI (*P* = 0.203).

**Conclusions:**

We found that structured imaging was beneficial for relapse detection during the first year after PCNSL diagnosis. A minor trend towards better survival after relapse was observed for patients who experienced relapse according to structured imaging.

## Background

Primary central nervous system lymphoma (PCNSL) is a rare, aggressive extranodal lymphomatous malignancy involving the central nervous system [1]. It has the highest growth rate of all central nervous system malignancies. For unknown reasons, its incidence seems to be increasing in Western countries [2–5]. An increasing number of patients with immunosuppression may explain some proportion of this, but an increase is also evident among immunocompetent people [2, 6].

Considering the high proliferation rate of PCNSL, rapid diagnosis and treatment initiation are essential for preserving patients’ cognitive function. The current standard of care for PCNSL includes intravenous high-dose methotrexate-based multiagent chemotherapy and rituximab, often combined with consolidative high-dose chemotherapy followed by autologous haematopoietic stem cell transplantation. An alternative to high-dose chemotherapy is whole-brain radiation for patients whose poor performance status prevents immunochemotherapeutic treatment or who do not respond to treatment [7–14]. In recent years, novel radiation therapy techniques, such as focal brain radiation and stereotactic radiation, have emerged for the treatment of brain tumours.

The prognosis of PCNSL patients is dismal [3, 15], and most relapses seem to occur within the first 2 years after diagnosis. Nevertheless, in contrast to other aggressive lymphomas, relapses may occur even 10 years after the primary diagnosis [16, 17]. Recent clinical trials with relatively short follow-up times and selected patient material have reported promising treatment results [10, 18]. However, this finding contrasts with most population-based reports, which have demonstrated that the prognosis of PCNSL patients is still poor, especially in older age groups [3, 5, 6, 15, 19–22].

There is no standard follow-up protocol for PCNSL patients. Generally, follow-up includes outpatient visits and structured imaging controls [23, 24]. Policies concerning outpatient visits and imaging frequency and the duration of follow-up vary among hospitals. Recent National Comprehensive Cancer Network guidelines recommend surveillance brain magnetic resonance imaging (MRI) every 3 months during the first 2 years after the completion of first-line treatment and every 6 months thereafter [25]. However, the optimal structured control imaging protocol for PCNSL follow-up is unknown [24, 26–29]. The aim of the present study was to determine the benefit of structured follow-up MRI studies and to determine the proportions of relapses that are detected based on regular imaging follow-up protocols and based on patients’ new symptoms.

## Methods

The data were gathered retrospectively from the Kuopio University Hospital, Oulu University Hospital, Tampere University Hospital, North Karelia Central Hospital, Hospital Nova of Central Finland, Mikkeli Central Hospital, Savonlinna Central Hospital and Lapland Hospital registries. The data were collected based on pathological diagnosis from neurosurgical tumour biopsy or spinal fluid cytology.

All PCNSL patients who were diagnosed between 2003 and 2020 regardless of the treatment protocol and who had available follow-up data were included in the study.

A total of 198 PCNSL patients (57.1% males) were included. Data concerning age, previous immunosuppression status, WHO performance score, histology, topography and morphology, cerebrospinal fluid (CSF) analysis, clinical disease presentation, relapses, and first-line and further given therapies were available for individual patients. The follow-up details included both structured and additional imaging studies and all outpatient and emergency visits and disease status at last follow-up date.

Primary PCNSL diagnosis was confirmed by neurosurgical tumour biopsy or positive spinal fluid cytology. Systemic lymphomas were excluded by whole-body computed tomography (CT) and bone marrow biopsy. Intraocular lymphoma was diagnosed by slit-lamp examination or from vitreous fluid cytology or flow cytometry.

Treatment was given as the standard of care based on the physician’s decision. During follow-up, the planned frequency of outpatient visits and routine imaging studies varied. Most commonly, patients were seen in outpatient clinics quarterly for the first 2 years and then biannually for 3 more years. Moreover, 2–4 routine MRI studies per year for 2 years and biannual MRI for 3 years was common practice.

Relapse was defined as disease recurrence after a complete response lasting for at least one month after therapy. The relapse diagnosis was performed mostly by MRI if the imaging results were considered adequate to initiate treatment. Some relapses were also diagnosed by CT, biopsy, CSF cytology and ophthalmologists.

The actualized imaging studies and visits were classified as a structured imaging study (sMRI) if it involved preplanned control imaging, an additional imaging study (aMRI) if based on the patient’s new neurological symptoms, a haematology or oncology outpatient visit (OV), an ophthalmology outpatient visit (OOV), or an additional ophthalmology outpatient visit (aOOV) due to the patient’s new ocular symptoms. Patients’ additional emergency or outpatient visits (AEs) were also evaluated. Most of the additional imaging studies (aMRIs) were ordered as an acute imaging from emergency departments.

Progression-free survival (PFS) was calculated from the date of diagnosis to the date of the first relapse, to the date of death from any cause, or to the last follow-up date, whichever occurred first. To determine the real impact of structured imaging on outcomes and avoid lead-time bias, we calculated survival for relapsed patients in two ways. First, we calculated overall survival (OS) according to the relapse detection method from the date of relapse to the date of death from any cause or to the last follow-up date (OS2). Next, we performed these same calculations starting from the date of the original diagnosis to the date of death from any cause or to the last follow-up date (OS1).

The data are shown as the means and ranges or frequencies and percentages. The Kaplan–Meier method was used to estimate survival rates with 95% confidence intervals (CIs) for outcome variables. Poisson regression was used to compare relapse counts between groups. P values < 0.05 were considered indicative of statistical significance. Statistical analyses were performed using IBM SPSS Statistics (version 27; IBM Corp, Armonk, NY).

## Results

The characteristics of the 198 patients included in this study are summarized in Table 1. The patients’ median age was 67 (13–89), and 60.1% (119) of patients were under the age of 70. Immunosuppression was noted in 7.6% (15) of the patients, one with HIV and one with organ transplantation and the rest with various autoimmune disorders and a history of immunosuppressive medication. Histological verification revealed diffuse large B-cell lymphoma in 95.5% (189) of the patients, T-cell lymphoma in 1.0% (2) and uncertainty in 1.5% (3) of the patients. Additionally, 2.0% (4) of the patients had indeterminate histology (2 patients with cerebrospinal fluid diagnosis without a brain biopsy and 2 patients with brain biopsy without available pathology reports in the hospital registry).

**Table 1:** Patient baseline demographics

Variable	No. = 198
Sex	
Female	85 (42.9%)
Male	113 (57.1%)
Age at diagnosis, years	
Median (range)	67 (13–89)
Age distribution	
< 70	119 (60.1%)
≥ 70	79 (39.9%)
WHO/ESOC performance status	
0	29 (14.6%)
1	60 (30.3%)
2	41 (20.7%)
3	51 (25.8%)
4	16 (8.1%)
Memorial Sloan Kettering Cancer Center (MSKCC) score	
Low-risk group: age ≤ 50	17 (8.6%)
Intermediate-risk group: age > 50 + Karnofsky Performance Scale (KPS) ≥ 70	117 (59.1%)
High-risk group: age > 50 + Karnofsky Performance Scale (KPS) < 70	64 (32.3%)
Immunosuppression	15 (7.6%)
HIV	1 (0.5%)
Organ transplantation	1 (0.5%)
Autoimmune disease	13 (6.6%)
Primary ocular involvement	21 (10.6%)
Isolated ocular disease	1 (0.5%)
Spinal fluid analysis	
Yes	150/198 (75.8%)
No	45/198 (24.2%)
Positive cytology, per patient:	
Malignant cytology, grade 5	6/89 (6.7%)
CSF blast cells	12/69 (17.4%)
Flow cytometry	9/51 (17.6%)
Histology	194 (98.0%)
Diffuse large B-cell lymphoma	189 (95.5%)
T-cell lymphoma	2 (1.0%)
Uncertain histology	3 (1.5%)
Unknown histology	4 (2.0%)
First-line treatment	
Yes/given	184 (92.9%)
Chemotherapy	171 (86.4%)
Bonn/Nordic protocol	74 (37.4%)
MATRIx	33 (16.7%)
BBBD	33 (16.7%)
Other methotrexate-based drugs	27 (13.6%)
Other combinations	4 (2.0%)
Autologous stem-cell transplantation	41 (20.7%)
Whole-brain radiation	38 (19.2%)
Initial treatment	31 (15.7%)
Consolidation therapy	7 (3.5%)
No treatment	14 (7.1%)
Mean follow-up, months (range)	39 (−3–229)
A total of 177 patients (89.4%)	
One patient was diagnosed via autopsy 3 months after death	

First-line treatment was given to 92.9% (184) of the patients; 86.4% (171) received chemotherapy, 20.7% (41) received autologous stem-cell transplantation, 15.7% (31) received whole-brain radiation as an initial treatment, and 3.5% (7) received consolidation therapy. The mean follow-up time was 39.2 months (−3–229). One patient was diagnosed via autopsy 3 months after death.

### Relapse pattern

Relapse was diagnosed in 71 patients (35.9%). The median time from diagnosis to the first relapse was 24.5 months (3–135).

Relapse was detected in 29.6% (21) of patients via sMRI, 57.7% (41) via aMRI, 2.8% (2) via OV, 4.2% (3) via OOV and 4.2% (3) via aOOV. In one patient, the method used to detect relapse was unknown. Six relapses were isolated ocular diseases. Only 19.4% (6/31) of the patients who received radiotherapy as first-line treatment underwent structured imaging (sMRI) follow-up protocol because of poor performance status.

Before the first relapse, patients had a mean of 2.04 (range 0–12) sMRIs, 0.84 (0–8) aMRIs, 3.69 (0–24) OVs, and 1.03 (0–8) AEs.

**Fig. 1:**
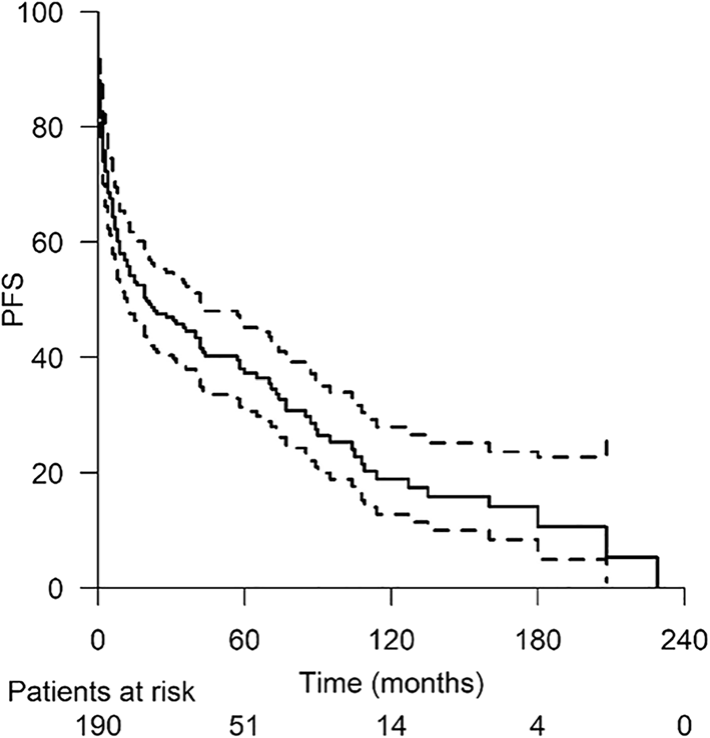
The median progression-free survival (PFS), months

Of the relapses discovered via MRI (62 patients), 66.1% (41) were discovered during the first 2 years after diagnosis, 46.8% (29) during the first year (0–12 months) and 19.4% (12) during the second year (13–24 months). However, 19.4% (12) of the relapses occurred during years 3 to 5 (25–60 months), and 14.5% (9) of the relapses occurred more than 5 years (60 months) after diagnosis. PFS is presented in Fig. 1.

The mean and cumulative numbers of sMRIs and aMRIs and relapses discovered during a certain time period after diagnosis are presented in Table 2.

**Table 2:** Mean and cumulative numbers of MRIs and relapses discovered during a certain time period

	sMRI	aMRI
0–12 months		
Mean	0.78 (0–4)	0.51 (0–4)
Cumulative, number of studies/patients	131/168	85/168
Relapses discovered	14 (48.3%)	15 (51.7%)
Number of MRIs needed to find a relapse	9.4 (131/14)	5.7 (85/15)
13–24 months		
Mean	0.81 (0–4)	0.20 (0–3)
Cumulative number of studies/patients	111/137	28/137
Relapses discovered	4 (33.3%)	8 (66.7%)
Number of MRIs needed to find a relapse	27.8 (111/4)	3.5 (28/8)
25–60 months		
Mean	0.81 (0–6)	0.48 (0–9)
Cumulative number of studies/patients	100/124	60/124
Relapses discovered	3 (25.0%)	9 (75.0%)
Number of MRIs needed to find a relapse	33.3 (100/3)	6.7 (60/9)
Over 60 months		
Mean	0.26 (0–5)	0.53 (0–6)
Cumulative number of studies/patients	25/98	52/98
Relapses discovered	0	9 (100.0%)
Number of MRIs needed to find a relapse		5.8 (52/9)

Second relapses were observed in 23 patients, 69.6% (16) of the relapses were discovered on aMRI, only 4.3% (1) on sMRI and 8.7% (2) in OV. Ophthalmologists identified 17.4% (4) of the patients with isolated ocular diseases.

In addition, 9 patients experienced subsequent relapses (range 3–5 total relapses per patient), and aMRI detected relapses in 55.6% (5) of the patients. Three relapses were isolated ocular disease.

### The benefit of structured MRI

**Fig. 2:**
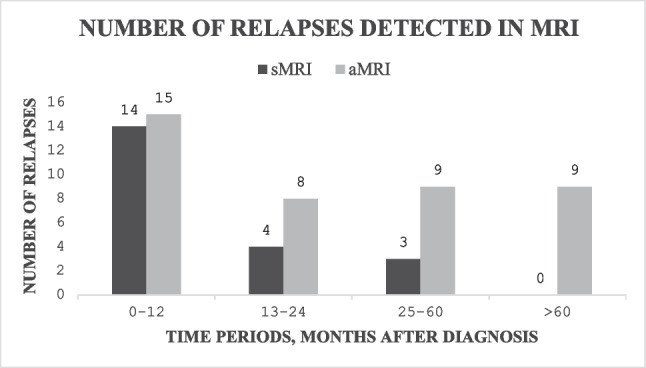
Number of relapses detected on sMRI and aMRI during certain time periods after diagnosis

During the first year (0–12 months) after diagnosis, 29 relapses were discovered; sMRI detected 48.3% (14) and aMRI 51.7% (15) of those relapses. During the second year (13–24 months), the respective rates were 33.3% (4) and 66.7% (8) of the 12 relapses. During years 3–5 (25–60 months), 12 relapses were discovered: 25.0% (3) were detected via sMRI, and 75.0% (9) were detected via aMRI. At more than 5 years (60 months) after diagnosis, all 9 relapses were detected via aMRI (Fig. 2).

To observe one relapse during the first year after diagnosis, 9.4 sMRI scans and 5.7 aMRI scans were needed. During the second year, the rates were 27.8 and 3.5, and during years three to five they were 33.3 and 6.7, respectively. More than 5 years after diagnosis, no single relapse was detected via sMRI, and the required number of aMRI studies for one relapse detection was 5.8.

When comparing the benefit of sMRI versus aMRI for relapse detection, most of the relapses were detected via aMRI at 13–24 months (*P* = 0.015) and 25–60 months (*P* = 0.023) after diagnosis.

Concerning subsequent relapses (32 total events), 65.6% (21) of the cases were detected via aMRI. Isolated ocular relapse was diagnosed in 21.9% (7) of the patients.

### The benefit of outpatient visits

Only 2 relapses were discovered in haematological or oncological outpatient visits (OVs) due to observed new symptoms of the patient which induced imaging. However, 6 relapses were revealed in ophthalmological outpatient visits (OOV and aOOV) in the slit-lamp examination.

### Survival

The median OS1 was 22.0 months. The 2-year OS1 was 50.0%, and the 5-year OS1 was 39.5%. The median OS2 was 3.0 months.

### The impact of the relapse detection method on survival

**Fig. 3:**
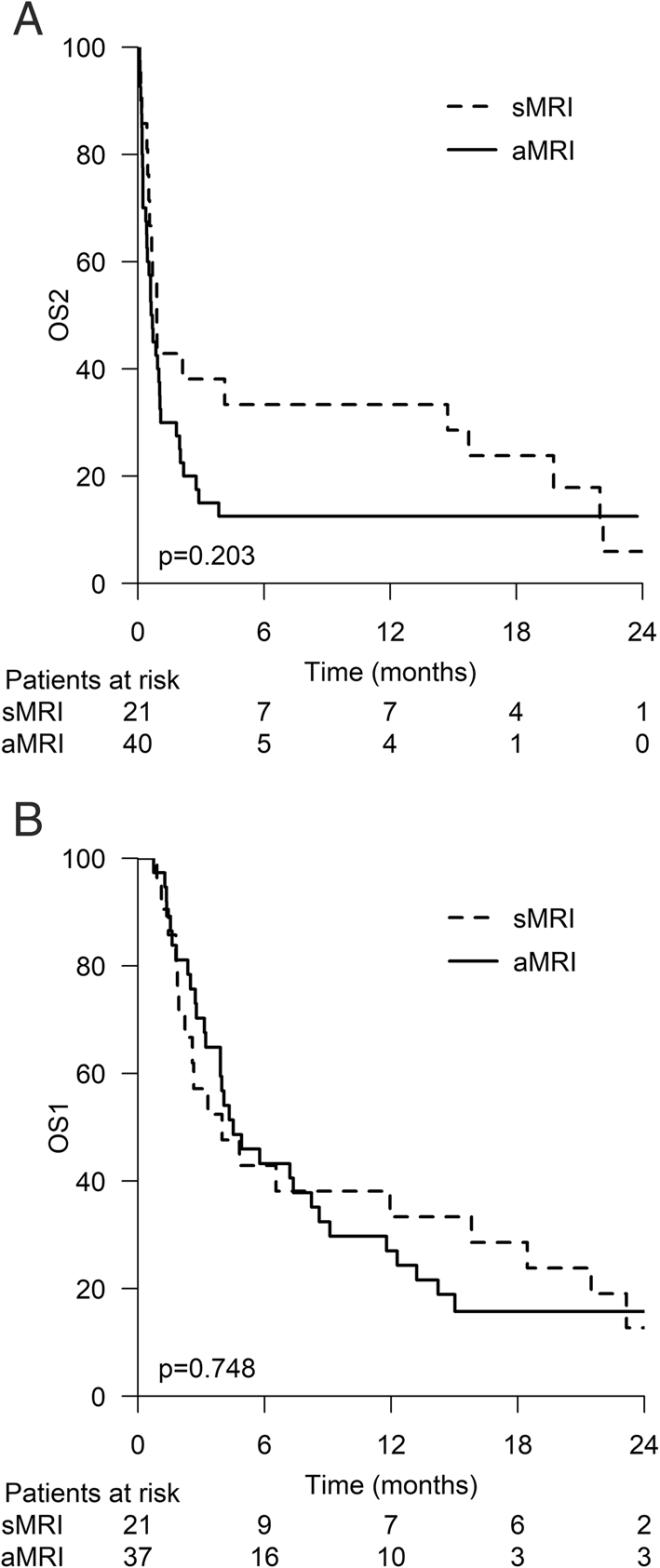
A) OS (months) from the date of the first relapse to the date of death from any cause or the last follow-up date (OS2) with respect to the relapse detection method: sMRI versus aMRI. Figure 3B) OS (months) from the date of diagnosis to the date of death from any cause or the last follow-up (OS1) with respect to the relapse detection method: sMRI versus aMRI

The median OS2 was 4.0 months (95% CI 2.5–5.5) for patients whose first relapse was detected via sMRI and 3.0 months (95% CI 1.2–4.8) for patients whose first relapse was detected via aMRI (*P* = 0.203) (Fig. 3A).

To rule out lead time bias, we also calculated the median OS1 of relapsing patients. It was 20.0 (95% CI 3.6–36.4) months for the patients whose first relapse was detected via sMRI and 22.0 (95% CI 13.1–30.9) months for those patients whose first relapse was detected via aMRI (*P* = 0.748) (Fig. 3B).

## Discussion

In the present study, we describe the modes of PCNSL relapse detection and assess the benefit of structured imaging protocols. To our knowledge, this is the first study to consider the timing of structural imaging benefit in PCNSL follow-up. We found a declining clinical benefit of preplanned imaging with longer follow-up time.

Limited data exist concerning the optimal PCNSL follow-up protocol and the benefit of structured MR imaging.

In the study by Fossard et al., which included 125 patients, 51% of whom experienced a relapse, only 10% of relapses were detected by planned brain imaging follow-up. The majority (80%) of relapses were diagnosed clinically based on new neurological symptoms. During the follow-up period, the median number of brain imaging studies was 7 (range 0–13). A total of 68 imaging studies were performed to detect one case of asymptomatic preclinical relapse. The difference in patient outcomes between asymptomatic and symptomatic relapse patients was not statistically significant; the one-year OS1 rates were 58% and 41%, respectively [27].

Mylam et al. reported an even greater relapse detection rate based on new clinical symptoms before planned brain imaging control. The study included 86 patients, 37% of whom experienced a relapse. The follow-up protocol was similar to that used in our study. Almost all (97%) of the relapses were detected by new clinical symptoms, and only one relapse (3%) was diagnosed by planned MRI scanning. Altogether, 189 routine MRI studies were performed to detect one case of preclinical relapse. The five-year OS1 was 58% [24].

A French study by Langner-Lemercier et al. included 563 patients, 45.5% of whom experienced a relapse/refractory disease. The median follow-up time was 9 months. Routine follow-up imaging detected 22.3% of the relapses. Compared with symptomatic patients, asymptomatic patients at first relapse had a better performance status and better OS2 (8.4 vs. 4.6 months) [26].

These earlier studies did not report results according to follow-up time, and to the best of our knowledge, this is the first study considering that aspect. In our study, more relapses were discovered on sMRI than in previous studies.

Concerning systemic aggressive lymphomas, data from the studies have not indicated the benefit of surveillance imaging in follow-up or its impact on survival [30].

We found that a structured imaging protocol (sMRI) was beneficial for relapse detection during the first year after diagnosis, when 48.3% of the relapses were detected via sMRI. The benefit of sMRI in relapse detection decreased over time when the relapse risk also decreased. Moreover, sMRI was not useful for subsequent relapse detection. However, in our study, the annual number of sMRIs performed for each patient decreased over time, which might have had an impact on the results.

Only 2 relapses were discovered in haematological or oncological outpatient visits (OVs). Considering this, it does not seem to be a very cost-effective method for relapse detection. However, outpatient visits are useful for patient well-being in follow-up protocols. Furthermore, in ophthalmological outpatient visits, slit-lamp examination revealed multiple relapses.

In line with multiple previous studies, PCNSL patient survival was poor (2–4). We discovered a minor trend toward a better second-line prognosis if relapse was observed on sMRI than on aMRI, but the difference was not statistically significant. In a retrospective setting, it is not possible to draw any conclusion regarding whether this is caused by earlier detection or whether patients with relapse discovered in an asymptomatic phase have a more indolent disease course in general.

During times of high pressure to control the utilization of health care resources, it is also important to evaluate whether this earlier relapse diagnosis benefits the patient. Naturally, when considering the potential benefits of routine imaging among PCNSL patients, patient comorbidities, age and previous treatment history should also be taken into consideration. If the patient has no potential effective therapy modalities remaining, nothing can be gained with earlier diagnosis either.

Our study has some limitations. In our retrospective real-world study, the patients’ follow-up protocols differed concerning imaging and outpatient visit frequencies between the participating hospitals. The data were analysed retrospectively, and some patients were lost to follow-up. Length time bias may have had impact on relapse detection and survival due to cases that are asymptomatically slowly progressing. Patients’ new neurological symptoms may have been underestimated and not taken as a prompt for contact and imaging. In other words, it is possible that asymptomatic patients were in fact symptomatic.

## Conclusions

In conclusion, the major clinical benefit of sMRI in relapse detection seems to be especially during the first year after diagnosis. However, considering the high relapse risk during the first 2 years after the end of therapy, we recommend that a structured MRI protocol could be used for 2 years after diagnosis for patients who are still able to receive curative PCNSL treatment. The optimal imaging interval during these first 2 years should be evaluated in future studies.

Even if routine imaging does not offer a survival benefit, we anticipate that relapse detection in the asymptomatic phase could help to preserve patients’ cognitive function. However, our retrospective dataset did not include neurocognitive surveillance data, so this conclusion should be regarded only as an assumption. Further studies are needed to compare survival, neurocognitive outcomes and quality of life between asymptomatic and symptomatic patients in terms of relapse.

## Data Availability

No datasets were generated or analysed during the current study.
